# A cause of circulatory collapse that should be considered following trauma

**DOI:** 10.1186/1755-7682-3-17

**Published:** 2010-08-06

**Authors:** Hesham R Omar, Engy M Helal

**Affiliations:** 1 Department of Cardiovascular medicine, Cairo University Hospital, Cairo, Egypt; 2 Emergency Department, Elagouza Hospital, Cairo, Egypt

## Abstract

Management of poly-trauma patients presenting to the emergency room is usually a challenging and formidable task. Two of the common problems seen in those patients are shock and neurological dysfunction. A huge differential for post-traumatic circulatory collapse exist and timely identification of the etiology is of utmost importance to avoid complications. In this report we are describing 2 cases presenting with circulatory collapse following trauma. The first case was a 29 year old female who presented after a motor vehicle accident fully conscious with severe hypotension and bradycardia. The second case presented with severe hemodynamic instability after falling at home. Physical examination of both patients revealed weakness in all 4 limbs and CT cervical spine revealed complete anterior sublaxation of C5 over C6 cervical vertebrae in the first case and partial sublaxation of C5 over C6 cervical vertebrae in the second case confirming that spinal cord injury is the likely cause for these hemodynamic alterations. A high index of suspicion for spinal cord injuries is therefore mandatory when managing a trauma patient presenting with quadriparesis and hemodynamic instability that is otherwise unexplained especially when the ensuing hypotension is associated with bradycardia instead of reflex tachycardia. Awareness of this cause of circulatory collapse is particularly important in the unconscious patient where eliciting sensory and motor deficits looking for spinal cord injury is not always feasible. Both patients were transferred to the intensive care unit and were maintained on epinephrine till becoming hemodynamically stable. The report aims to sensitize readers to this cause of post-traumatic circulatory collapse.

## Introduction

There is a long list of causes for hemodynamic instability following trauma. These were best elucidated by a simple mnemonic described by Ho[1]. Spinal cord injury is one of these causes and should be suspected when other more common factors are not present and especially when a trauma victim presents with quadriparesis in addition to hypotension and bradycardia. 2 clinical scenarios demonstrating circulatory collapse following trauma will be discussed.

## Case #1

A 29 year old woman with no medical history of significance presented to the emergency room after a motor vehicle accident. Initial assessment revealed a fully conscious patient who is pale, cold and sweaty. Vital signs revealed a blood pressure of 60/30 mmHg, pulse of 40/minutes, respiratory rate of 30/minute and a temperature of 37°C. Chest examination revealed equal air entry on both sides with areas of coarse crepitations and sonorous ronchi more over the right lung which we attributed to pulmonary contusions. Cardiac examination and the jugular venous pressure were normal. Neurological examination revealed a Glasgow coma score of 15/15. Motor system examination revealed upper limbs weakness grade 1/5 and lower limbs weakness grade 0/5 associated with atonia and areflexia. Sensory system examination revealed absent sensations in all four limbs and the patient was incontinent to urine and stool.

There was no evidence of external bleeding or internal bleeding as proved by abdominal ultrasound, no clinical evidence of tension pneumothorax or massive pleural effusion and no orthopedic fractures. The patient was properly sedated ruling out reflex neurogenic shock from pain as the possible etiology. Chest X-ray and computed tomography revealed bilateral lung contusions more on the right side with no evidence of pneumothorax or pleural effusion. CT brain was normal. Arterial blood gases revealed PO_2 _of 64 and a SO_2 _of 93% on room air and the patient was maintained on 5 liters of oxygen/minute through a nasal cannula raising her saturation to 99%. Intravenous fluid resuscitation with normal saline and atropine were commenced without any improvement in her hemodynamic parameters.

**Figure 1:**
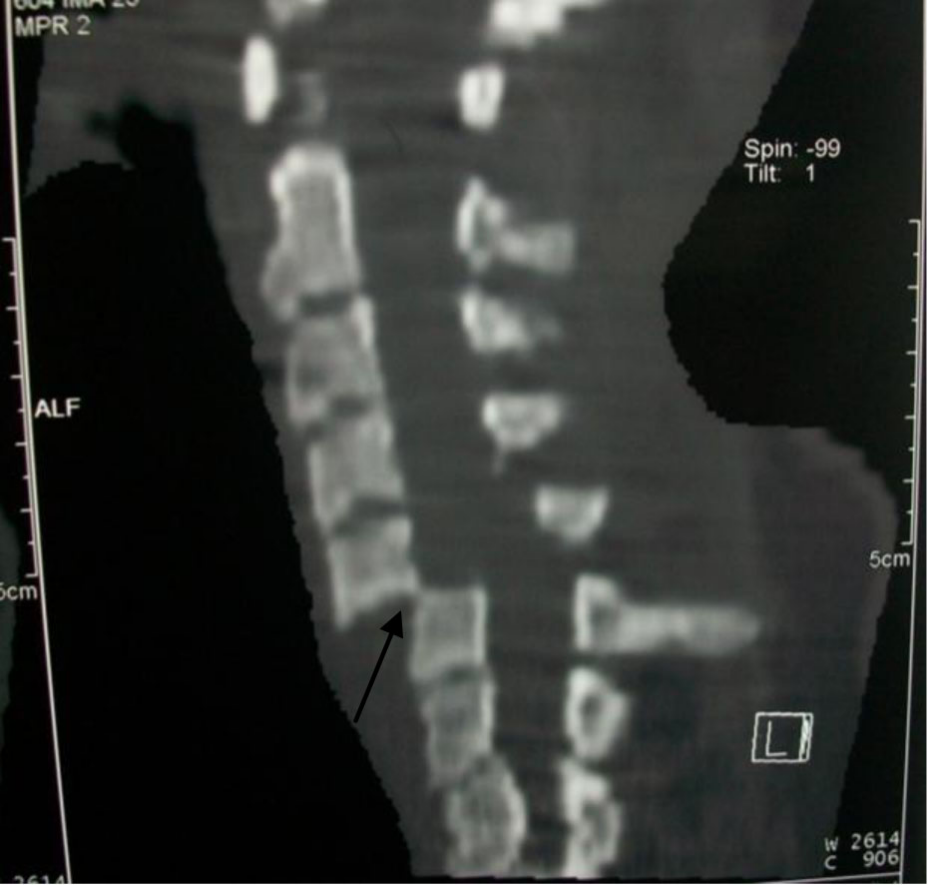
**CT scan of the cervical spine revealing complete anterior sublaxation (C5 over C6 cervical vertebrae)**.

The quadriparesis, hypotension, bradycardia and absence of an obvious cause explaining her hemodynamic instability suggested that spinal cord injury is the underlying etiology which can be responsible for both the neurologic deficits and circulatory collapse. To further confirm the diagnosis, CT of the cervical spine revealed complete sublaxation of C5 over C6 cervical vertebra as shown in figure 1.

The patient was then transferred to the ICU for proper monitoring and correction of her hemodynamics. She was maintained on intravenous adrenaline therapy which dramatically improved her hemodynamic parameters and was sent to the operating room for fixation of C5 and C6 vertebrae. Over the next weeks in her ICU stay, the patient did not show any signs of neurological recovery and later succumbed to chest infection and complications of prolonged recumbency.

## Case # 2

A 70 year old man with a medical history of diabetes mellitus, hypertension and renal impairment presented to the emergency room after he slipped at his home. On admission, the patient was hypotensive with a BP of 60/40 and bradycardic with a heart rate of 40/minute. Neurological examination revealed a fully conscious patient with a Glasgow coma score of 15/15, complete paralysis of both lower limbs (grade 0/5) and severe weakness of both upper limbs (grade 2/5). There was also complete anesthesia in all 4 limbs.

The patient's respiratory pattern was slow and shallow. Chest examination revealed equal air entry on both sides with normal vesicular breathing. Arterial blood gases revealed a PH of 7.31, PCO2 of 51 mmHg, PO2 of 120 mmHg, HCO3 of 26 and oxygen saturation of 98% on 10 liters of oxygen supplied by a face mask. There was no evidence of external or internal bleeding, no clinical evidence of cardiac tamponade or contusion and no evidence of massive hemothorax or tension pneumothorax that can otherwise explain the patient's hemodynamic instability.

**Figure 2:**
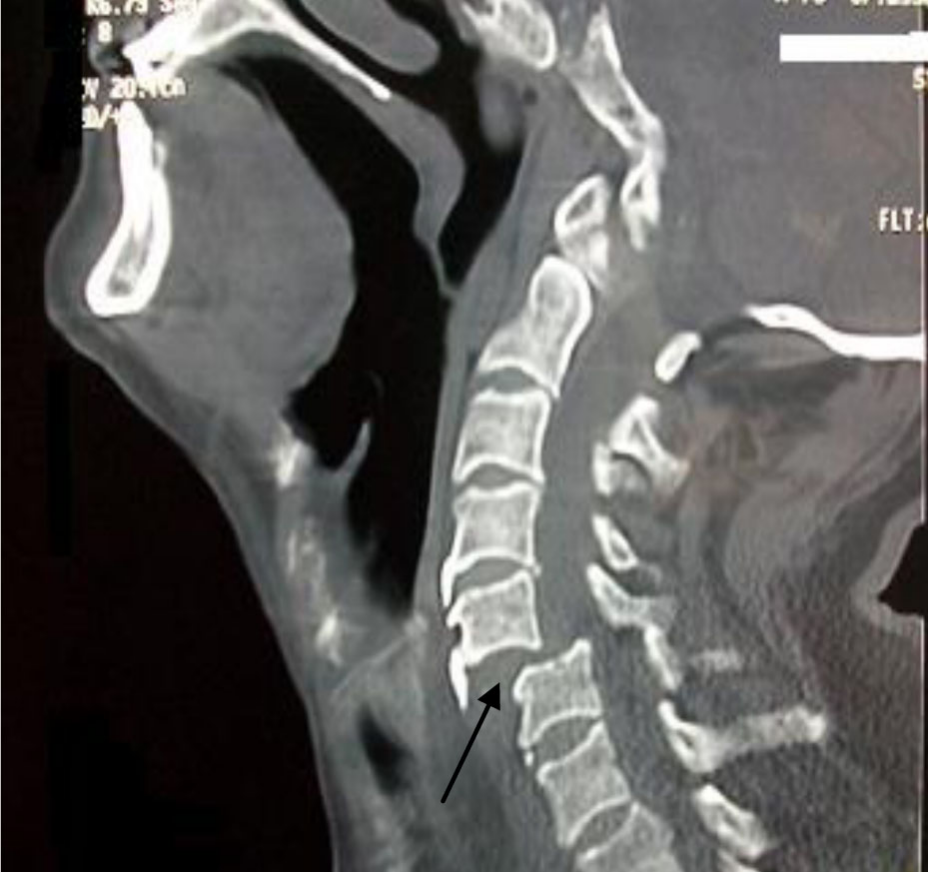
**CT scan of cervical spine revealing partial anterior sublaxation (C5 over C6 cervical vertebrae)**.

CT cervical spine revealed partial sublaxation of C5 over C6 cervical vertebra as shown in figure 2. The patient was transferred to the ICU for proper monitoring and control of his hemodynamics where intravenous epinephrine was administered. After hemodynamic stability ensued, the dislocation was fixed by plate and screws. During his hospital course, the patient was mechanically ventilated because of respiratory failure and CO_2 _retention due to diaphragmatic paralysis.

## Discussion

Circulatory collapse following trauma has numerous causes. An exhaustive search for the underlying etiology is mandatory and the management is usually a challenging task. Common etiologies include bleeding, cardiac complications in the form of tamponade, cardiac contusions, aortic injury, myocardial ischemia and chest complications including massive hemothorax or tension pneumothorax. Timely identification of the cause is of utmost importance to avoid complications.

Of these causes and which is often unrecognized is the spinal cord injury. Acute spinal cord injury causes total loss of sympathetic innervations and may be complicated by life threatening hemodynamic alterations. Neurogenic shock with severe hypotension and bradycardia can be a consequence of spinal cord injury and occurs hours to months after the initial insult. This usually resolves in 3-6 weeks. These complications usually occur if the cord is injured at the cervical level. In a study conducted by Guly et. al., the incidence of neurogenic shock in cervical cord injuries was 19.3% in comparison with thoracic and lumbar cord injuries which was 7% and 3% respectively[2].

Spinal cord damage is usually due to cord contusion, compression or stretch rather than complete transection. The developing hypotension may aggravate the ischemia of the spinal cord which can lead to further damage of the fragile neuronal tissues[3,4]. Early recognition and appropriate management of this complication is therefore mandatory to improve perfusion to the injured and distorted spinal cord [5,6] and to minimize secondary injury.

Whereas most of the factors (bleeding, cardiac causes, chest causes) causing hypotension are associated with reflex tachycardia, in patients with spinal cord injury hypotension is characteristically associated with bradycardia due to interruption of sympathetic fibers. A high index of suspicion for the presence of cord injury is therefore mandatory in any trauma victim presenting with hypotension and bradycardia.

In addition to neurogenic shock, the acute phase of spinal cord injury is also associated with ''spinal shock''. Mistakenly, these terms are usually used interchangeably inspite of being 2 distinct conditions. Neurogenic shock is characterized by hemodynamic alterations that occur in the heart rate and blood pressure following spinal cord injury whereas spinal shock is characterized by a marked reduction of sensory, motor or reflex function of the spinal cord below the level of injury[7,8].

In conclusion, acute spinal cord injury is a diagnosis that should be suspected in any trauma victim presenting to the emergency room if the patient is experiencing hypotension and bradycardia that is not otherwise explained. This report aims to sensitize readers to this often neglected cause of circulatory collapse following trauma.

## Competing interests

The authors declare that they have no competing interests.

## Authors' contributions

HO and EH have made substantial contributions to conception and design, drafted the manuscript, revised it critically for important intellectual content and gave final approval of the version to be published.

## Consent

Written informed consent was obtained from the patients for publication of this case report. A copy of the written consent is available for review by the Editor-in-Chief of this journal
